# Supplementary material for: Understanding Patterns of the Gut Microbiome May Contribute to the Early Detection and Prevention of Type 2 Diabetes Mellitus: A Systematic Review
Source: Microorganisms. 2025 Jan 10;13(1):134. doi: 10.3390/microorganisms13010134 (PMC11767308; doi:10.3390/microorganisms13010134)
Supplement: Supplementary file 1 [file microorganisms-13-00134-s001.zip › microorganisms-3390212-supplementary.pdf]

Table S1: Bias assessment criteria

JBI Checklist/Study	Were the criteria for inclusion in the sample clearly defined?	Were the study subjects and the setting described in detail?	Was the exposure measured in a valid and reliable way?	Were objective, standard criteria used for measurement of the condition?	Were confounding factors identified?	Were strategies to deal with confounding factors stated?	Were the outcomes measured in a valid and reliable way?	Was appropriate statistical analysis used?	Overall	Risk
Diener et.al.	1	1	1	1	1	1	1	1	8	Low risk of bias
Maskarinec et.al.	1	1	1	1	1	0	1	1	7	Low risk of bias
Shen et.al	1	1	1	1	1	1	1	1	8	Low risk of bias
Li Qian	1	1	1	1	0	0	1	1	6	Moderate risk of bias
Karlsson	1	1	1	1	0	0	1	1	6	Moderate risk of bias
Ruuskanen	1	1	1	1	1	0	1	1	7	Low risk of bias
Wang	1	1	1	1	1	1	1	1	8	Low risk of bias
Vals-Delgado	1	1	1	1	1	1	1	1	8	Low risk of bias
Hao Wu	1	1	1	1	1	0	1	1	7	Low risk of bias
Allin	1	1	1	1	0	0	1	1	6	Moderate risk of bias
Kwan	1	1	1	1	1	1	1	1	8	Low risk of bias
Chen Z	1	1	1	1	1	1	1	1	8	Low risk of bias
Kitten AK	1	1	1	1	0	0	1	1	6	Moderate risk of bias
Larsen et al	1	1	1	1	0	0	1	1	6	Moderate risk of bias
Sedighi et al.	1	1	1	1	1	0	1	1	7	Moderate risk of bias
Ahmad et.al.	1	1	1	1	0	1	1	1	7	Low risk of bias
Neri-Rosario et al	0	0	1	1	0	0	1	1	4	High risk of bias
Carrizales-Sánchez	1	1	1	1	0	0	1	1	6	Moderate risk of bias
Guo et.al	1	1	1	1	1	1	1	1	8	Low risk of bias
Chang et.al	1	1	1	1	1	0	1	1	7	Low risk of bias
Wu et.al	1	1	1	0	0	0	1	1	5	High risk of bias

Criteria used to rank the risk of bias:	
≤49%=high risk of bias	
50-69%=Moderate risk of bias	
above 70% = low risk of Bias	
Coded answer numerical	
Yes	1
No	0

Table S2: Selected studies overview

	Paper 1	Paper 2	Paper 3	Paper 4	Paper 5	Paper 6	Paper 7
Author	Diener et.al.	Maskarinec et.al.	Shen et.al	Li Qian	Karlsson	Ruuskanen	Wang
Date of publication	2021	2020	2021	2020	2013	2022	2022
Country /population	Mexico	USA	China	China	China	Finland	China
Type of study	cohort study /cross sectiona;	Retrospective coho	retrospective cohort for m	cohort	cohort	Cohort observational	Populational longitu
Significant changes in microbiome in T2D?	Yes	Yes	Yes	Yes	Yes	Yes	Yes
T2D sample size	48	315	229	30		432	123
Control group size (normal glucose)	214	765	50251	30		5140	2772
Prediabetic sample size	42	518	462				
Total size of the study	405	1756	53970	60	145	5572	2895
16 s rRNA sequencing type	Ilumia						Ilumia
Primers	V4	V1-V3		V4 V5			V4
Bioinformatic tool for data processing	QIIME						
Alpha diversity Shannon index	Yes	Yes			yes and Chao index		
Alpha diversity higher or lower in T2D?	Lower	Lower		Lower			
Genera positively associated with T2D (unhealthy)	*Escherichia, Veillonella*	*Escherichia-Shigella, Lachnospiraceae*	*Dorea, Blautia, Anaerostipes, Eggerthella , Ruminococcus*	Dorea	Faecalibacterium, Dorea , Fusobacterium ,Lactobacillus gasseri, Streptococcus mutans, Clostridium clostridioforme	*Lachnospiraceae, Clostridium (ruminococcus , Egghertella , coprococcus, blautia, anaerostipes)*	Dorea
Genra negatively associated with T2DM (healthy)	*Blautia, Anaerostipes*	Actinobacteria, Firmicutes	*Bifidobacterium, Bacteroides, Faecalibacterium, Akkermansia and Roseburia*	Bifidobacterium , Akkermansia	Parabacteroides , Streptococcus, Bifidobacterium and Akkermansia , Oscillospira, Roseburia	*Alistipes , sutterella*	Erysipelatoclostridium, Parabacteroides ,Paraprevotella, [Ruminococcus], Dialister
Type of treatment	*Metformin*						

	Paper 8	Paper 9	Paper 10	Paper 11	Paper 12	Paper 13	Paper 14
Author	Vals-Delgado	Hao Wu	Allin	Kwan	Chen Z	Kitten AK	Larsen et al
Date of publication	2022	2020	2018	2022	2021	2021	2010
Country /population	Spain	Sweden	Denmark	Mexico	Netherlands	USA	Denmark
Type of study	cross sectional	discoivery cohort	case control stud	Cohort	populational cro	cross section	cross section
Significant changes in microbiome in T2D?	Yes	Yes	Yes	Yes	Yes	Yes	Yes
T2D sample size	107	46		75	193	14	
Control group size (normal glucose)		523	134	141	1056	23	18
Prediabetic sample size	355	297	134		1146		18
Total size of the study	462	569	268	216	2166	37	36
16 s rRNA sequencing type	Ilumia		illumia			Illumia	
Primers	V4		V4			V4	V4
Bioinformatic tool for data processing						NA	
Alpha diversity Shannon index	Yes		yes		yes	yes	
Alpha diversity higher or lower in T2D?	No difference		lower		Lower	no significant difference	
Genera positively associated with T2D (unhealthy)	*Prevotella , Sutterella, Paraprevotella ,Dialister, Lactobacillus,Anaerostipes*	*Enterobacterales , Enterobacteriaceae , Escherichia/Shigella , Gammaproeteobacteria , proteobacteria , Fusicatenibacter , Lactobacillus ,*	Dorea, [Ruminococcus], Sutterella and Streptococcus ,Faecalibacterium	Enterobacteriaceae, Escherichia-Shigella, Prevotella	Acetitomaculum, Christensenellaceae R7 group,Marvinbryantia,Ruminococcaceae	Proteobacteria,Verrucomicrobia	Bacteroidetes, Proteobacteria,Prevotella spp., Lactobacillus spp.
Genra negatively associated with T2DM (healthy)	Akkermansia, Faecalibacterium , Clostridiaceae, Porphyromonadaceae , Ruminococcaceae, Odoribacter, Butyricimonas, Barnesiella, Parabacteroides, Bacteroidetes, Collinsella, Lachnospira	*Faecalibacterium* sp p., *Clostridium spp.*, *Alistipes spp.*, *Pseudoflavonifractor spp.*, and *Oscillibacter* ,*Clostridiales bacterium*, *Flavonifractor plautii*, *Coprococcus eutactus*, *Alistipes obesi*, *and Intestinimonas butyriciproducens;*	Clostridium , Akkermansia muciniphila,	Faecalibacterium prausnitzii, Peptostreptococcaceae, Romboutsia and Clostridium sensu stricto 1	Clostridiums ensustricto1 ,Peptostreptococcaceae,Intestinibacter, Romboutsia	Bacteroidetes,Bifidobacterium genus,Firmicutes phylum, Roseburia genus ,Streptococcus genus	Firmicutes, Clostridia sp p.
Type of treatment						Metformin	

	Paper 15	Paper 16	Paper 17	Paper 18	Paper 19	Paper 20	Paper 21	Totals
Author	Sedighi et al	Ahmad et.al.	Neri-Rosario et al	Carrizales-Sánchez	Guo et.al	Chang et.al	Wu et.al	
Date of publication	2017	2019	2023	2023	2024	2024	2010	
Country /population	Iran	Pakistan	Mexico	Mexico	China	Taiwan	China	
Type of study	cohort	cohort	Machine learning associ	cohort	cohort	cohort	cohort	
Significant changes in microbiome in T2D?	Yes	Yes	Yes	Yes	Yes	Yes	Yes	
T2D sample size	18	40		21	93		16	1800
Control group size (normal glucose)				20	25	60	12	61184
Prediabetic sample size	18	20				57		2770
Total size of the study	36	60	410	41	118	117	28	15369
16 s rRNA sequencing type				Illumina	Illumina	Illumina		
Primers		V3 V4		V3 V4	V3V4	V3 V4	V3	
Bioinformatic tool for data processing								
Alpha diversity Shannon index				yes				
Alpha diversity higher or lower in T2D?				higher				
Genera positively associated with T2D (unhealthy)	*Lactobacillus group*	*Firmicutes , Clostridia , Negativicutes , Eubacterium coprostanoligenes group, Dialister , Allisonella , Bacillus sporothermodurans , and Staphylococcus sp. SV3*	*Enterobacterales , Enterobacteriaceae , Escherichia/Shigella , Gammaproeteobacteria , proteobacteria , Fusicatenibacter , Lactobacillus , Dielma , and Allisonella*	*Faecalibacterium, Prevotella, Dorea , and Lactobacillus*	*Bacteroides , Parabacteroides , Phasolarctobacterium, [Ruminococcus], Dorea, Ruminococcus, Collinsella*	*Bacteroides , Phascolarctobacterium , Parabacteroides , and Paraprevotella*	*Clostridium leptum*	
Genra negatively associated with T2DM (healthy)	*Bifidobacterium* group	*Bacteroidetes*, *Verrucomicrobia*, *Proteobacteria*, *Elusimicrobia*, and *Prevotella_9*	*Lachnospiracea UCG.004*, *Holdemanella*, *Ruminococcus_1*, and *Anaerostipes*	*[Ruminococcus]*, *Succinivibrio*, and *Bacteroides*	*Blautia*, *[Ruminococcus]*, *Dorea*, *Ruminococcus*, *Faecalibacterium*, *Bifidobacterium*, *Collinsella*	*Blautia*, *Faecalibacterium*, *Bifidobacterium*, *Clostridium*, *Anaerostipes*, *Mediterraneibacter*, *Streptococcus*, *Eggerthella*, *Butyricicoccus*	*Bacteroides vulgatus* and *Bifidobacterium genus*	
Type of treatment				Metformin				

Table S3: Bacteria reported in both groups across the studies.

Bacteria reported in both groups across the studies			
	**Positively associated with T2DM**	**Negatively associated with T2DM (healthy)**	**Comment**
Blautia	Shen et.al and Ruuskanen et.al	Diener et.al.	Ruuskanen et.al. conducted large observational cohort of 5572 participants, however T2D sample size was relatively low as most of the participants observed in this study belonged to control group. Nevertheless, the increase in *Balutia* has also been confirmed in Shen at.al. study , which is a multi-ethnic study with a sample size of 53970 participants. The study by Shen et.al included Mexican population (n=405) which might not be exact representation of ethnical diversities.
Collinsella	Guo et.al	Vals-Delgado et.al.	Vals-Delgado reported the species as healthy. However, the results are based on relatively low sample size. Interestingly study by Vals-Delgado et.al the T2DM group is 3 times smaller than a control group, whilst in Gu et.al study the ratio of T2D:Control was 3.72 ,on the contrary to Vals-Delgado which may explain the discrepant results of these two reports.
Dialister	Vals-Delgado et.al. and Ahmad et.al.	Wang et.al.	Ahmad et,al. compared T2D versus prediabetic and also seen the statistically significant difference in these bacteria, suggesting positive association with T2DM.
Dorea	Shen et.al , Li Qian, Karlsson et.al, Wang et.al. , Allin et.al,Carrizales-Sánchez et.al; Guo et.al	Guo et.al	Positively associated with T2DM in 7 independent studies and only 1 study found it on the contrary.
Paraprevotella	Vals-Delgado and Chang et.al	Wang et.al.	2 studies of total sample size 579 found the species to be positively associated with T2D, whilst Wang et al. study of n=2895 found the species negatively correlated. These differences observed might be due to geographic locations/ethnicity.
Prevotella	Vals-Delgado, Kwan, Larsen et al , Carrizales-Sánchez et.al.	Ahmad et.al.	Only one study of a low sample size showed negative association with T2DM.
Proteobacteria	Kitten AK, 14 Larsen et al , Ahmad et.al., Neri-Rosario et al	Ruuskanen et.al.	Only 1 study found these bacteria healthy, with 4 different cohorts reporting positive correlation with T2DM
Sutterella	Vals-Delgado and Allin et.al reported both as unhealthy species positively associated with 2TDM, with much smaller sample size of 462 and 268 participants	Kitten AK et.al	Very low sample size , results reported as percentages rather than differential abundance therefore questionable. Also *Akkermansia* belongs to genus *Verrucomicrobia* ,but it is well recognized for its beneficial effects on glusose balance , therefore one needs to look more into lower taxonomic level to make better conclusions.
Anaerostipes	Shen et.al , Ruuskanen, Vals-Delgado	Diener et.al. ;Neri-Rosario et.al ; Chang et.al	
Faecalibacterium	Karlsson et.al;Allin et.al; Carrizales-Sánchez et.al.	Shen et.al ;Vals-Delgado et.al ; Hao Wu et.al ;11 Kwan et.al ; Guo et.al; Chang et.al	
